# Cross-Sectional Survey on *Toxoplasma gondii* Infection in Cattle, Sheep, and Goats in Algeria: Seroprevalence and Risk Factors

**DOI:** 10.3390/vetsci6030063

**Published:** 2019-07-10

**Authors:** Mohamed-Cherif Abdallah, Miroud Kamel, Benfodil Karima, Ansel Samir, Khelef Djamel, Kaidi Rachid, Ait-Oudhia Khatima

**Affiliations:** 1 Laboratoire Hygiène Alimentaire et Système Assurance Qualité (Hasaq), Ecole Nationale Supérieure Vétérinaire d’Alger. Rue Issad. Oued Smar., Bab Ezzouar, Algeria; 2 Laboratoire Epidémio-Surveillance, Santé, Productions et Reproduction, Expérimentation et Thérapie Cellulaire des Animaux Domestiques et Sauvages (ESSPRETCADS), Institut des Sciences Vétérinaires, Université Chadli Bendjedid El-Tarf, ElTarf 36000, Algeria; 3 Laboratoire Biotechnologie et Reproduction Animale (LBRA), Institut des Sciences Vétérinaires Blida, Ouled Yaïch, Algeria

**Keywords:** seroprevalence, *T. gondii*, cattle, sheep, goat, elisa, Algeria

## Abstract

A cross-sectional study aimed at assessing the seroprevalence and identifying the risk factors for *Toxoplasma gondii* infection in cattle, sheep, and goats in eight provinces located in two main Algerian agro-ecological zones was carried out from October 2015 to March 2018. Blood sera from 4074 animals of both sexes were tested for the presence of anti-*T. gondii* IgG antibodies, using the indirect, enzyme-linked, immunosorbent assay technique (ELISA). Moreover, to identify the potential risk factors of *T. gondii* infection, a survey through a breeders’ questionnaires was conducted. Nearly one-fourth of the total number of animals tested (1024/4074)—i.e., 25.1%—were seropositive. The seroprevalence in cattle, sheep, and goats was 28.7%, 25.6%, and 11.9%, respectively. The area, sex, age, and herd size were identified as risk factors for *T. gondii* infection. Higher seropositivity rates were recorded in cows and goats (odds ratio (OR) = 1.63 and 6.4), in old animals (cattle, OR = 2.1; sheep, OR = 1.9; and goat, OR = 3.9), and in small size herds (cattle, OR = 2.5; sheep, OR = 1.9; goat, OR = 2.2). In conclusion, there is widespread *T. gondii* infection in cattle, sheep, and goats in these two strategic agricultural areas. The identification of the risk factors determines the type of measures and strategies to be undertaken to reduce, control, and prevent *T. gondii* infection in domestic animals, and thereby reduce human infection.

## 1. Introduction

*Toxoplasma gondii* is an obligate intracellular protozoan that causes widespread infection in humans and many other warm-blooded animal species (mammals and birds); it has adverse effects on public health and animal production. While many animals serve as intermediate hosts, only domestic cats are definitive hosts. The parasite is transmitted to these hosts through contaminated meat, milk, and water. Such contamination can arise with oocyst-contaminated foodstuffs, which is an important route for the contamination of farm animals [1,2].

Ingestion of ecologically robust stages (sporozoites in oocysts), consumption of raw or undercooked meat, or meat products containing tachyzoites or bradyzoites is the main transmission route of *Toxoplasma* to humans [3,4]. Most infections in humans are asymptomatic, but serious complications may occur following congenital *Toxoplasma* infection, such as abortion, stillbirth, mortality, and hydrocephalus in newborns, or retinochoroidal lesions leading to chronic ocular disease and lymphadenopathy, retinitis, or encephalitis in immunocompromised individuals [5].

*Toxoplasma* infection has been reported in wild and domestic animals. In food-producing animals, however, sheep and goats are more often infected than cattle or chickens [3,6]. Sheep and goats have been reported as a major source of infection in several countries [1,6]. Recently, tachyzoites of *T. gondii* have also been detected in the milk of several intermediate hosts, including camels [2,7].

*T. gondii* infection in ruminants as a major cause of abortions and stillbirths [8], and brings about significant economic losses to the global sheep, goat, and cattle industry [9,10]. It gives rise to a wide variety of non-specific (fever and dyspnoea) and specific (fever, depression, lethargy, vomiting, diarrhea, chorioretinitis, and lymphadenopathy) symptoms [11,12].

The control of *T. gondii* infection in cattle, sheep, and goats is therefore important, not only for the efficient breeding of domestic animals, but also for public health.

In Algeria, although the prophylaxis of congenital toxoplasmosis is part of a national surveillance program of pregnant women that provides medical treatment of toxoplasmic seroconversions or active toxoplasmosis, the human seroprevalence reported is still quite high (51.6%) [13].

There are very few studies on toxoplasmosis prevalence in domestic animals, despite its omnipresence in Algeria. As a consequence, the status of the *T. gondii* infection is not yet established and is poorly understood. This is what motivated us to carry out a cross-sectional survey on cattle, sheep, and goats in several sensitive parts of Algeria, in order to assess the seroprevalence of *T. gondii* infection in food-producing domestic ruminants, as well as to identify the main risk factors that are associated with the infection.

## 2. Materials and Methods

### 2.1. Study Area

With an area of 2,381,741 square kilometres, Algeria is the largest country in Africa and the Mediterranean basin. Its southern part is mainly occupied by the Sahara. To the north, Atlas Tell forms with the Saharan Atlas; further south, two sets of parallel reliefs point east, between which are inserted vast plains and uplands. In fact, by type of livestock, it there are 26.88 million sheep, 4.90 million goats, and 1.90 million cattle. Sheep farming accounts for almost 80% of the total number of national herds.

The study area was chosen on purpose to represent two sensitive agroecological zones (high- and lowlands) of central Algeria. These two zones are distributed in 12 provinces called *wilayates*, in the highlands (Boumerdes, Tizi-Ouzou, Bouira, and Setif), midlands (Tiaret, M’sila, El-Bayadh, and Djelfa), and lowlands (Algiers, Medea, Saida, and Laghouat).

Livestock production is widely distributed in the region, and the number of herds is high. Semi-extensive production is predominant for cattle, sheep, and goats, and is characterized by housing in the winter months until early spring, at the time of delivery, and extensive pasture the rest of the year.

### 2.2. Animals and Samples

A cross-sectional study design was used. Different age and sex groups of cattle, sheep, and goats were included for this study. The study was conducted from October 2015 to March 2018. Serological investigation was used to detect anti-*T. gondii* antibodies from blood serum collected from animals in the districts under study. Since there was no previous expected prevalence in the area, sample size was calculated according to Thrusfield, using an expected prevalence of 50%, a desired precision of 5%, and with 95% level of confidence [14]. Hence, the sample size was 384 for each species. Due to the fact that the goat population at the study area was very limited in number, 478 goats, 2144 sheep, and 1452 blood samples of cattle sera were subjected to analysis. In total, 4074 samples were analyzed.

Three age groups were established: <2 years, 2–5 years, and ˃5 years. Individuals were randomly selected within herds, to a maximum of 30 sheep, 15 cattle, and 10 goats per herd. The herd is considered seropositive when at least one animal from the same herd had anti-*T. gondii* antibodies. Finally, 1452 cattle, 2144 sheep, and 478 goats from 95, 70, and 47 herds, respectively, were surveyed.

Additional data collected for each sampled animal included gender, breed, husbandry system (semi-intensive or extensive), and geographic origin, which is a well-known risk factor for *T. gondii* infection are retained for this study [15].

Blood samples were collected from the jugular or tail vein, depending on the animal species, in 10 mL vacutainer tubes with no anti-coagulants or preservatives, as approved by the National Consultative ethnical committee for life sciences and health. The samples, after sitting overnight at room temperature, were centrifuged at 3000 rpm for 10 min. The sera were stored at -20 °C in 1.5 mL Eppendorf tubes until analysis.

### 2.3. Serologic Testing

The sera were tested for the presence of anti-*T. gondii* antibodies using an Toxoplasmosis Indirect ELISA Multi-species kit (ID Screen, ID.VET. Innovative Diagnostics, Montpellier, France), according to manufacturer’s instructions. The sensitivity of this ELISA test reaches 100%, whereas specificity was determined to be of 96% (manufacturer’s data).

The results were expressed as optical density (OD); absorbance was read at 450 nm with an EL-800 ELISA Plate reader (Biotek Instruments Inc., USA). The 96-well plate was coated with P30 *T. gondii* antigens, and the antigen–antibody complex formed with the help of the peroxidase conjugate, which was added later. Positive and negative controls were provided by the manufacturer and were used to validate each test. The samples were considered positive if they had a value ≥50%, doubtful for values between 40% and 50%, and negative if ≤40%. This percentage was calculated as follows: percentage of positivity = 100 × OD of the sample/OD of the PC (OD: Optic Density; PC: Positive Control). Information about the sensitivity and specificity of this ELISA test (100% and 97.8%, respectively) was provided by the manufacturer.

### 2.4. Statistical Analysis

The data were recorded and coded using a Microsoft Excel spreadsheet, and analysed using the SPSS software (SPSS Inc., IBM Corporation, Version 22, Chicago, IL, USA). The seroprevalence was calculated by dividing the number of animals positive to anti-*T. gondii* antibodies by the total number of animals tested. The relationship of risk factors with the dependent variable was primarily assessed using cross tabulation. Univariable logistic regression analysis was performed, and the strength of the association between risk factors and *T. gondii* infection were evaluated with Chi-square tests. Multivariate logistic regression was used for all variables showing a moderate statistical significance (*p* ˂ 0.05) in the univariate analysis. The logistic regression model was developed in a stepwise forward approach, using a likelihood ratio test at each step (*p* < 0.05 to enter and *p* > 0.10 to exit). Model fit was assessed with the Hosmer and Lemeshow goodness-of-fit test. All statistical analyses were performed using the statistical software SPSS version 22.0 (SPSS Inc., Chicago, IL, USA). In all analyses, two-tailed *p* values <0.05 were considered as statistically significant.

## 3. Results

The present seroepidemiological survey was based on field samples. It reflects the importance of studies on *Toxoplasma* on a regional basis from ruminant species in Algeria.

At least one seropositive animal was detected in 204 out of the 212 herds tested, which gave an estimated seroprevalence at the herd level of 96.2% (95% CI: 93.7%–98.8%).

Over the 204 farms that were tested (at least one animal), 93/95 (97.8%; 95% CI: 95%–100%), 70/70 (100%; 95% CI: 100%–100%), and 41/74 (87.2%; 95% CI: 77.7%–96.8%) concerned cattle, sheep, and goats, respectively (Table 1). We considered these farms as positive for *T. gondii* infection. It is important, though, to indicate that all the animals making up the herds were not blood-sampled.

The individual seroprevalence at the animal level in the study area (12 wilayates of central Algeria), adjusted for sampling sizes and for test sensitivity and specificity, was 25.1% (1024/4074, 95% CI: 23.8%–26.5%).

The *T. gondii* seropositivity rate was 28.7% (418/1452; 95% CI: 26.5%–31.1%) in cattle, 25.6% (549/2144; 95% CI: 23.8%–27.4%) in sheep, and 11.92% (57/478; 95% CI: 9%–14.8%) in goats (Table 1).

### 3.1. Cattle

Table 2 summarises the results of the univariate analysis of individual-level risk factors for *T. gondii* seroprevalence in cattle.

Four factors were associated with seropositivity against *T. gondii*: gender (*p* ˂ 0.0000), age group (*p* ˂ 0.00001), herd size (*p* ˂ 0.00001), and geographical area and provinces (*p* ˂ 0.009). The individual seroprevalence was higher in females (32.9%) than in males (19.5%), in cattle more than 5 years old (52.4%) than those less than 2 years old (14.5%), in small (50.13%) than large herd sizes (20.8%), and in centrally located provinces (Table 2).

**Figure 1:**
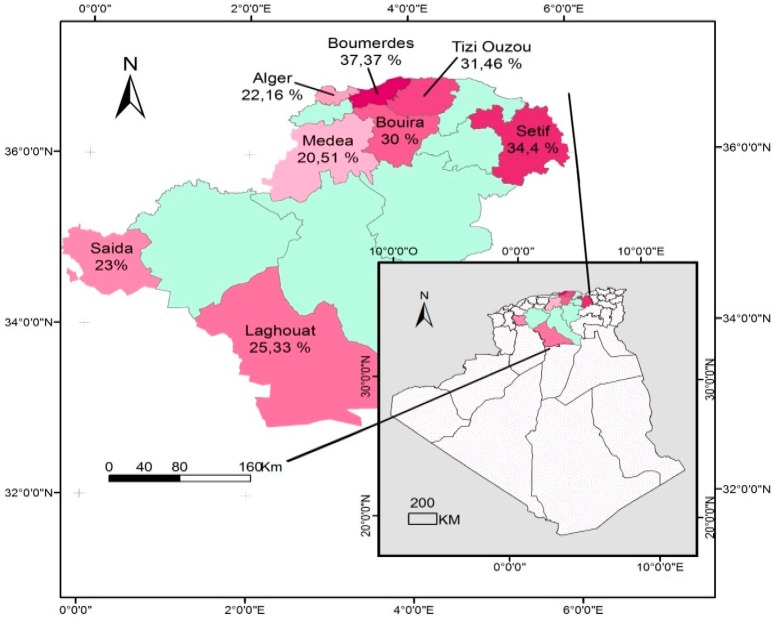
Distribution of cattle seroprevalence of *Toxoplasma gondii* infection in Algeria.

The cattle seroprevalence recorded in the 08 provinces involved ranged from 20.5% (Medea) to 37.3% (Boumerdes), as seen in Table 2 and Figure 1. The breed, rearing system, and water source were not significantly associated with seropositivity.

The four risk factors investigated were simultaneously analysed via a logistic regression model to determine their relative contributions to *T. gondii* seropositivity. In the final model, the three following risk factors were found to be associated with *T. gondii* infection: farm location, age group, and herd size. A two-fold increased risk of infection was seen for cattle from small-herd-size farms (OR = 2.5; CI: 1.19–5.23; *p* = 0.004), and for the age group >5 years old (OR = 2.1; CI: 1.02–5.11; *p* = 0.024). Moreover, cattle reared in the highland areas of Algeria (Boumerdes, Tizi-Ouzou, Bouira, and Setif) had significantly (OR = 2.8; CI: 1.25–6.13; *p* = 0.014) higher risk of infection with *T. gondii* than those reared in the lowlands (Algiers, Medea, Saida, and Laghouat).

### 3.2. Sheep and Goats

The results of the univariate and multivariate analysis of the individual-level risk factors for *T. gondii* seroprevalence in sheep are summarised in Table 3, and those for goats are in Table 4.

**Figure 2:**
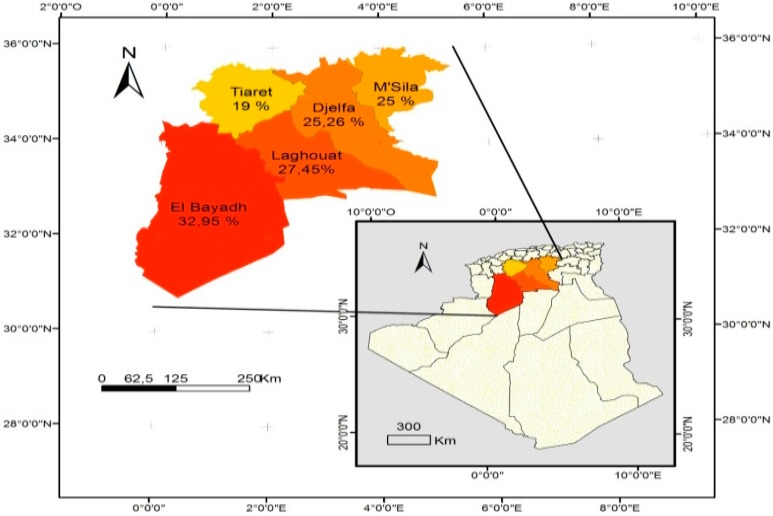
Distribution of the sheep seroprevalence of *Toxoplasma gondii* infection in Algeria.

**Figure 3:**
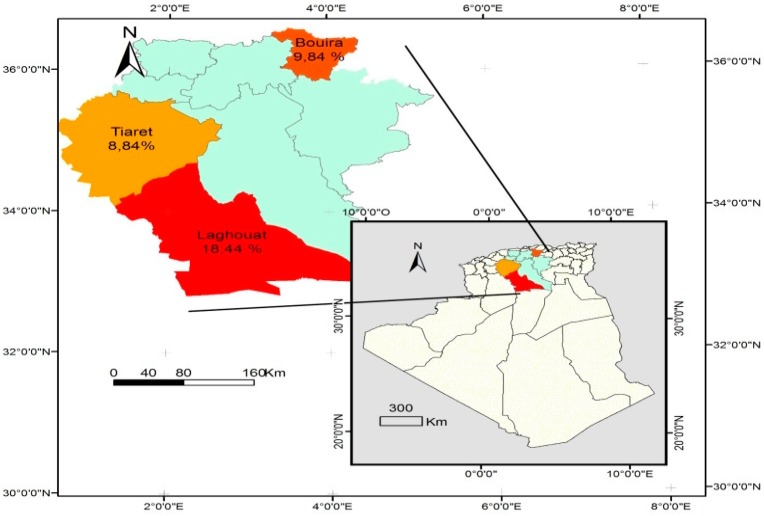
Distribution of goat seroprevalence of *Toxoplasma gondii* infection in Algeria.

In addition, the seroprevalence recorded in small ruminants in wilayates forming the study area ranged from 19% in Tiaret to 32.9% in El bayadh (Table 3 and Figure 2) in sheep, and from 8.8% in Tiaret to 18.4% in Laghouat (Table 4 and Figure 3) in goats.

Factors associated with seropositivity in univariate analysis were age group (*p* ˂ 0.00001 for sheep and goats), female gender in goats (*p* ˂ 0.00001), herd size (*p* ˂ 0.00001 and 0.0016, respectively for sheep and goats), and region (*p* ˂ 0.0004 and 0.0169, respectively, for sheep and goats).

Results of multivariate logistic regression analysis indicates that the potential risk factors related to age group, gender, and herd size revealed that the likelihood of *T. gondii* infection was higher in adult sheep (OR = 1.93; CI: 1.12–3.36; *p* = 0.001) and in small herds (OR = 1.95; CI: 1.11–3.44; *p* = 0.002), when compared with young sheep and large herd sizes, respectively. The likelihood of *T. gondii* infection was two times higher in adult goats >5 years old (OR = 3.9; CI: 1.63–9.41; *p* = 0.015) and six times higher in female goats (OR = 6.08; CI: 2.2–14.6), with a two-fold increase from small compared to large farms (OR = 1.95; CI: 1.11–3.44; *p* = 0.002). However, there was no difference in the prevalence of infected animals from farms with an extensive rearing system and semi-intensive, or according to water source.

Figure 1, Figure 2, and Figure 3 show the seroprevalence distribution of *T. gondii* infection in cattle, sheep, and goats, respectively, in the study area.

## 4. Discussion

Humans around the world are highly dependent on domestic animals for many reasons, including needs for meat, milk, and fat [16].

The present study provided a better understanding of *Toxoplasma* infection, and revealed widespread *T. gondii* infection in food-producing domestic ruminants in Algeria. The presence of anti-*Toxoplasma* antibodies has been confirmed in animal farms in all the provinces involved in the study, indicating extensive contamination by the parasite.

When considering the Algerian people’s habit of eating undercooked grilled beef, sheep, and goat meat, the risk of contracting toxoplasmosis is to be highly considered.

The prevalence of *T. gondii* infection in domestic ruminants has gained increasing interest in recent years, due to the animals’ role in the spread of the parasite, either through direct contact or via consumption of animal meat products [8,12,17,18].

The high seroprevalence in cattle (97.89%), sheep (100%), and goats (87.23%) recorded in the present study could reflect the high level of environmental contamination and widespread distribution of the parasite.

These seroprevalences recorded in domestic ruminants are higher than those reported previously: 58.89% and 84.61% in Algeria [19], 45.17% in Tanzania [20], and 70.48% in Ethiopia [21]. This seroprevalence could reflect the heavy agricultural soil contamination with oocysts, as has been stated in many studies [17,22]. These agreements can be attributed to grazing systems where many herds graze daily. As a result, the risk of contact with contaminated feed and pasture during the grazing season was high in herds of animals.

The results of this first region-wide survey on *T. gondii* infection in cattle, sheep, and goats determined high seroprevalences in domestic food-producing ruminants (28.7%, 25.6%, and 11.9% in cattle, sheep, and goats, respectively).

The seroprevalences obtained for cattle (28.7%) is higher than those reported previously, which were 3.9% [19], 4.4% [23], and 15.2% [24] in Algeria; this shows that *T. gondii* infection has increased considerably. However, some of the discrepancy between the reported results could be due to (1) the small sample size of animals, which may impair the representativity and objectivity of data; (2) the wide geographic area concerned or covered; (3) management practices (traditional, semi-intensive, and extensive); and (4) the serological methods used for detecting the infection. In this study, and in another recently published paper [25], the ELISA technique was used, whereas the results reported earlier in Algeria were obtained through an indirect immunofluorescence test (IFAT) [19] and microscopic agglutination test (MAT) [23]. This difference may be due to the sensitivity and specificity of the different serological methods applied [6,12].

Throughout the world, data on *T. gondii* seroprevalence in cattle are considerably different. They vary from 0% to 99% [15], including from 2% to 92% in Europe [3] and from 10% to 37% in North Africa [12]. The seroprevalence of *T. gondii* infection in cattle reported in the present study is more or less similar to that reported (27.4%) in Libya [26], but it is higher than that reported (2.68%) in Brazil [27], in Central Ethiopia (6.6%) [28], in Indonesia (9%) [29], in East Ethiopia (10.4%) [30], in Vietnam (10.5%) [31], in Tanzania (13%) [32], in Iran (15.77%) [33], and in Thailand (22.3%) [34]. Other studies have reported a lower value: 30% in the Netherlands [35], 32% in Sudan [36], and 51.96% in Brazil [37]. Such data should be analyzed with caution, since geographical variations occur not only between different countries, but also within countries. In addition, the comparison of data reported in different countries requires the use of standardized tests, procedures, and appropriate size sampling. These data are not directly comparable, because of the variability in the sampling strategy, the type of testing methods used, and the protocol of analysis adopted.

Although some studies have reported a high seropositivity in cattle, *Toxoplasma* is considered to be far less infective to cattle. In this species, the clinical signs are usually not observed in naturally infected animals, and the parasite has very rarely been detected in tissues of an adult cows [38] or in aborted fetuses [39].

In sheep, data on *T. gondii* infection vary widely. The observed value in the present study (25.6%) is quite similar with those previously reported in Morocco (27.6%) [40] and in Libya (26.2%) [26], but it is slightly higher than those recorded in many other countries, such as central Ethiopia (22.9%) [27], Morocco (20.8%) [41], Tunisia (19%) [42], Pakistan (11.1%) [43], Algeria (11.6% and 8.3%) [19,44], and northeastern China (3.0%) [45]. Lower seroprevalence values have, however, been observed in other parts of the world, such as in Brazil (29.41% and 32.9%) [46,47], Ethiopia (31.5% and 33.7%) [21,30], Italy (33.3%) [48], Tunisia (40.2%) [49], and Egypt (52.7%) [50], as well as in Nazareth, Ethiopia (56%) [51].

In goats, the *T. gondii* infection seroprevalence that we observed was close to that recorded (11.2%) in Pakistan [43], Central Ethiopia (11.6%) [28], northern America (12.1%) [52], and Algeria (13.2%) [19], but was higher than that observed in Morocco (8.5%) [41], South Africa (4.3%) [53], and India (3.8%) [54]. Other studies reported higher seroprevalences than ours, such as those reported in Ethiopia (25.9%) [55], Thailand (27.9%) [56], Tunisia (34%) [49], Pakistan (41.8%) [57], Egypt (41.7% and 44.3%) [58,59], Libya (50%) [26], and Southern Ethiopia (55.18%) [60].

The variations in the overall seroprevalence observed in the current study, as well as those mentioned above, could be due to the access level of the small ruminants to contaminated feed and water, climatic variation, and the diagnostic techniques used [61,62].

The logistic regression indicated that, in addition to farm location, small herd size, female gender, and adulthood are the main risk factors for cattle infection.

Moreover, cattle raised in highland areas had significantly higher risk of *T. gondii* infection than those raised in lowland ones. This observation was also made in almost all studies throughout the world [21,43,57,60,63].

This discrepancy between authors could be due to the variation in temperature and humidity in the areas of studies, since it has been reported that environment influences the epidemiology of toxoplasmosis [3,61,64]. Algerian highlands have a very high moisture content, which increases the chance of oocyst survival in the environment and the contact probability with contaminated sources, thus leasing to higher seroprevalences [2,30,65]. On the contrary, a dry climate has a negative impact on the survival and epidemiological distribution of the parasite [2,60].

The size of the herd, and notably when it is small, is considered as the major risk factor in the present study, no matter the species considered. In Algeria, and particularly in the areas of study, the type of farm management adopted could be at the origin of such an observation. In Algeria, small herds are the ones managed traditionally, because (1) the livestock’s aliment is widely accessible to cats; (2) the animals’ grazing is frequent, and the transition from intensive to extensive and vice versa was done daily; and (3) the lack of zoo-hygienic measures, including feeding organizing, cleaning, etc. These were extensively reviewed by Tenter et al. [3], Klun et al. [66], and Dubey et al. [2].

The relationship between the age of the 1452 cattle, 2144 sheep, and 478 goats sampled and *T. gondii* seroprevalence was studied. This latter was significantly higher in the adults (52.43% cattle, 31.5% sheep, and 31.48% goats) than in the young animals (14.51% cattle, 20.66% sheep, and 9.12% goats). The multivariable logistic regression analysis showed that the likelihood of acquiring infection was higher in the adults (cattle: OR = 2.1, CI: 1.02–5.11, *p* = 0.024; sheep: OR = 1.93, CI: 0.89–3.33, *p* = 0.001; goats: OR = 3.9, CI: 1.81–6.32; *p* = 0.002) than in the young animals. These findings are similar to those of Jittapalapong et al. [56]; Teshale et al. [51], and Tilahun et al. [30], who reported a low prevalence in young animals and a high one in adults. Seroprevalence of the *T. gondii* antibody has been found to increase with age, no matter the animal species. This could be due to a longer exposure of the adults to *T. gondii* infection [2]. Animals that have lived longer are more likely to be *T. gondii* parasite sources [12,64,67].

With regard to the sex risk factor, the study showed that the seroprevalence of anti-*T. gondii* antibody is higher in cattle females (33%) than in cattle males (19.5%). The multivariable logistic regression analysis revealed that the likelihood of acquiring infection was higher in females (OR = 1.63, CI: 1.01–3.16, *p* = 0.018) than in males of this species. This is in agreement with what was reported by Clementino et al. [47] and Zewdu et al. [21], but not by Silva et al. [37] and Lashari and Tasawar [36], who observed the opposite—a higher seroprevalence in males than in females. Other authors, however, have reported no significant differences between the two genders [64]. The increased sensitivity of females may be associated with a lower immunological resistance during certain periods of their lives [68], such as the stress of lactation and pregnancy, which causes an immunosuppression that renders them more liable to *T. gondii* infection [30,69].

In our study, the presence of cats was not investigated as a risk factor, since it is partly ubiquitous in almost all farms studied or those located nearby. The presence of resident or stray cats may explain the high prevalence of *T. gondii*-specific antibodies observed in this study, due to oocyst clearance and environmental contamination. The significant of cats as a reservoir of *T. gondii* infection is confirmed by the finding of a high infection rate in domestic ruminants, which strongly supports the high seroprevalence of toxoplasmosis in women observed in Algeria [13,70].

## 5. Conclusions

*T. gondii* infection has been found to be widespread among cattle, sheep, and goats reared in the studied areas. The risk factors identified are area, sex, age, and herd size; these are important to set the control and prophylactic measures and strategies to reduce *T. gondii* infection in domestic animals, and thereby in humans. Thus, the higher seroprevalence encountered in these animal species used as a food source reveals the potential risk of *T. gondii* infection presented to people through consumption of their meat. Therefore, the population should be sensitized through education on the modes of transmission and prevention of *T. gondii* infection, and further study should be conducted to explore the impact of the disease on food animal production

## Figures and Tables

**Table 1:** Seroprevalence of *Toxoplasma gondii* infection in domestic cattle, sheep, and goats from Algeria.

Species	Animal Level				Herd Level			
	*n*	No. of positive	Percentage (%)	95% CI	n*	No. of positive	Percentage (%)	95% CI
Cattle	1452	418	28.7	26.5–31.1	95	93	97.8	95–100
Sheep	2144	549	25.6	23.8–27.4	70	70	100	100–100
Goat	478	57	11.9	9–14.8	47	41	87.2	77.7–96.8
**Total**	4074	1024	25.1	23.8–26.5	212	204	96.2	93.7–98.8

*n*: number of sampled animals; No.: number; n*: number of herds involved; CI: confidence interval.

**Table 2:** Analysis of risk factors related to *T. gondii* seroprevalence in cattle at the animal level.

Variables		*n*	No. of Positive	Percentage (%)	95% CI	*p-*Value	OR (95% CI)	*p-*Value
Age group (years)	˂2	448	65	14.51	11.55–18.07	0.00001 *		0.024 *
	2–5	737	213	28.90	25.74–32.28			
	˃5	267	140	52.43	46.45–58.35		2.1 (1.02–5.11)	
Gender	Male	446	87	19.5	15.83–23.18	0.00000 *		0.0018 *
	Female	1006	331	33	30.17–35.97		1.63 (1.01-3.16)	
Breed	Imported	953	275	28.85	26–31.73	0.98524		
	Local	499	143	28.66	24.7–32.62			
Rearing system	Semi-Intensive	840	241	28.7	25.6–31.7	0.97020		
	Extensive	612	177	28.92	25.3–32.5			
Herd size (head)	Small (˂20)	387	194	50.13	45.17–55.08	0.00001 *	2.5 (1.2–5.32)	0.014 *
	Medium (20–50)	720	152	21.11	18.29–24.24			
	Large (˃50)	345	72	20.87	16.91–25.47			
Water Source	Well	871	265	30.42	27.46–33.56	0.10361		
	Lake/river	581	153	26.33	22.92–30.06			
Area (farm location)	Alger	167	37	21.15	15.86–28.45	0.00911 *		
	Boumerdes	303	95	31.46	22.22–36.69			
	Tizi-Ouzou	190	71	37.37	30.59–44.25			
	Bouira	190	57	30	23.48–36.52			
	Medea	78	16	20.51	11.55–29.45			
	Setif	125	43	34.4	26.07–42.73			
	Saida	100	23	23	14.75–31.25			
	Laghouat	300	76	25.33	20.41–30.25			

*n*: number of animals sampled; No.: number; CI: confidence interval; OR: odds ratio; *: *p* < 0.05.

**Table 3:** Analysis of risk factors related to *T. gondii* seroprevalence in sheep at animal level (*n =* 2144).

Variables		*n*	No. of Positive	Percentage (%)	95% CI	*p-*Value	OR (95% CI)	*p-*Value
Age group (years)	˂2	779	161	20.66	17.8–23.5	0.00001 *		0.001 *
	2–5	838	222	26.5	23.5–29.5			
	˃5	527	166	31.5	27.5–35.5		1.93 (1.12–3.33)	
Gender	Male	922	218	23.64	20.9–26.3	0.07872		
	Female	1222	331	27.08	24.6–29.6			
Rearing System	Semi-Intensive	623	149	23.91	20.6–27.3	0.27446		
	Extensive	1521	400	26.3	24.1–28.5			
Herd size (head)	Small (˂20)	510	183	35.88	31.84–40.14	0.00001 *	1.95 (1.10–3.43)	0.002 *
	Medium (20–50)	639	153	23.94	20.8–27.4			
	Large (˃50)	995	213	21.4	18.97–24.06			
water source	Well	1100	298	27.09	24.55–29.8	0.10595		
	Lakes	1044	251	24.04	21.55–26.73			
Province	M’Sila	364	91	25	20.6–29.4	0.00041 *		
	Tiaret	542	103	19	15.7–22.31			
	El-Bayadh	4331	142	32.95	28.51–37.38			
	Djelfa	388	98	25.26	20.93–29.58			
	Laghouat	419	115	27.45	23.17–31.72			

*n*: number of animals tested; No.: number; CI: confidence interval; OR: odds ratio; *: significant.

**Table 4:** Analysis of risk factors related to *T. gondii* seroprevalence in goats at animal level (*n =* 478).

Variables		*n*	No. of Positive	Percentage (%)	95% CI	*p-*Value	OR (95% CI)	*p-*Value
Age group (years)	˂2	263	24	9.12	6.12–13.22	0.00001 *	-	
	2–5	161	16	9.93	6.12–15.53		-	
	˃5	54	17	31.48	20.68–44.75		3.9 (1.81–6.32)	0.002
Gender	Male	64	7	10.93	5.4–20.9	0.00000 *	-	
	Female	414	50	12.07	9.28–15.57		-	
Rearing System	Semi-Intensive	96	13	13.54	8.08–21.8	0.7108	-	
	Extensive	382	44	11.51	8.69–15.11		-	
Herd size (head)	Small (˂20)	205	35	17.07	12.54–22.82	0.0106 *	1.95 (1.10–3.43)	0.002
	Medium (20–50)	182	15	8.24	5.06–13.15			
	Large (˃50)	91	7	7.69	3.78–15.04			
Water source	Well	178	21	11.79	7.8–17.36	0.94740		
	Lakes	300	36	12	8.8–16.17			
Region/Province	Bouira	122	12	9.83	4.55–15.12	0.01693 *		
	Tiaret	215	19	8.84	5.04–12.63			
	Laghouat	141	26	18.44	12.04–24.84			

*n*: number of animals tested; No.: number; CI: confidence interval; OR: odds ratio; *: significant.
